# Development and Validation of Spectrophotometric Methods for the Estimation of Mesalamine in Tablet Dosage Forms

**DOI:** 10.4103/0975-1483.66789

**Published:** 2010

**Authors:** KM Patel, CN Patel, B Panigrahi, AS Parikh, HN Patel

**Affiliations:** Department of Analysis, Shri Sarvajanik Pharmacy College, Near Arvind Baug, Mehsana - 384 001, Gujarat, India

**Keywords:** Bratton-Marshall reagent, Gibb’s reagent, mesalamine, P-dimethylaminobenzaldeyde, visible spectroscopy

## Abstract

Three simple and sensitive visible spectrophotometric methods (A, B, and C) have been developed for the quantitative estimation of mesalamine in bulk drug and pharmaceutical dosage forms. Methods were based on the formation of colored chromogens, which were measured at 552 nm, 440 nm, and 494 nm, respectively. The results obtained with the proposed methods were found to be unsatisfactory with the labeled amounts when the tablet dosage forms were analyzed.

## INTRODUCTION

Mesalamine is chemically, 5-amino-2-hydroxybenzoic acid, which is used as a gastrointestinal anti-inflammatory drug for the treatment of inflammatory bowel diseases[1] and active ulcerative proctitis.[2,3] It is not official in any pharmacopoeia. The literature shows that mass spectroscopy[4] and high performance liquid chromatography[5] (HPLC) have been reported for determination of mesalamine. Spectrophotometric analytical reports are not found for its quantitative estimation in bulk drug and tablet dosage forms in literature. In the present study, new, simple, and selective UV Spectrophotometric methods were elaborated for the determination of mesalamine in commercial dosage forms.

### Development and validation of a HPLC-ESI-MS/MS method for the determination of 5-aminosalicylic acid and its major metabolite N-acetyl-5-aminosalicylic acid in human plasma.[4]

A new HPLC method for the determination of 5-aminosalicylic acid (5-ASA) and N-acetyl-5-aminosalicylic acid (N-Ac-5-ASA) in human plasma was developed and validated. The plasma samples were analyzed after protein precipitation with methanol and the two analytes were separated using a C18 column, with the mobile phase composed of 17.5 mmol/L acetic acid (pH 3.3) : acetonitrile = 85:15 (v/v) at 0.2 mL/min flow rate. 4-ASA and N-Ac-4-ASA were used as internal standards. Selective detection was performed by tandem mass spectrometry with electrospray source, operating in negative ionization mode, and in multiple reaction monitoring acquisition (m/z 152-->108 for 5-ASA; m/z 194-->150 and 194-->107 for N-Ac-5-ASA). The method was applied to evaluate the pharmacokinetics of 5-ASA after a single oral dose administration of this compound (1200 mg) to 24 healthy volunteers. The mean maximum concentration levels were 680 ng/mL for 5-ASA and 1240 ng/mL for N-Ac-5-ASA, and the kinetic profiles were in agreement with previous studies.

### High-performance liquid-chromatographic determination of 5-aminosalicylic acid and its metabolites in blood plasma.[5]

A new HPLC bioanalytical method for the determination of 5-ASA and its metabolites in blood plasma was developed and validated. The sample preparation step consisted of the deproteination of plasma by HClO(4) and the above-mentioned derivatization of ASAs, followed by liquid–liquid extraction of all N-acyl-ASA-derivatives. Chromatographic analyses were performed on a 250 – 4 mm column containing Purospher RP-18 e, 5 microm (Merck, Darmstadt, Germany) with a precolumn (4 – 4 mm). The column effluent was monitored using both UV photodiode-array (lambda = 313 nm) and fluorescence detectors (lambda (exc.) = 300 nm / lambda(emiss.) = 406 nm) in tandem. The identity of individual N-acyl-ASAs in the extracts from biometrics was verified by characteristic UV-spectra and by HPLC / MS experiments. The whole analysis lasted for 23 minutes at a flow rate of 1 ml min (-1). LLOQ (LOD) was estimated at 126 (20) pmol ml (-1) of plasma for N-acetyl-5-ASA and 318 (50) pmol ml (-1) of plasma for N-propionyl-5-ASA. The validated HPLC method was applied to the pharmacokinetic studies of mesalazine in humans and animals.

## MATERIALS AND METHODS

Three simple and sensitive visible spectrophotometric methods (A, B, and C) have been developed for the quantitative estimation of mesalamine by using Bratton-Marshall reagent (BMR), paradimethylaminobenzaldehyde (PDAB), and Gibb’s reagent at room temperature.

### Method A

**Figure 1:**
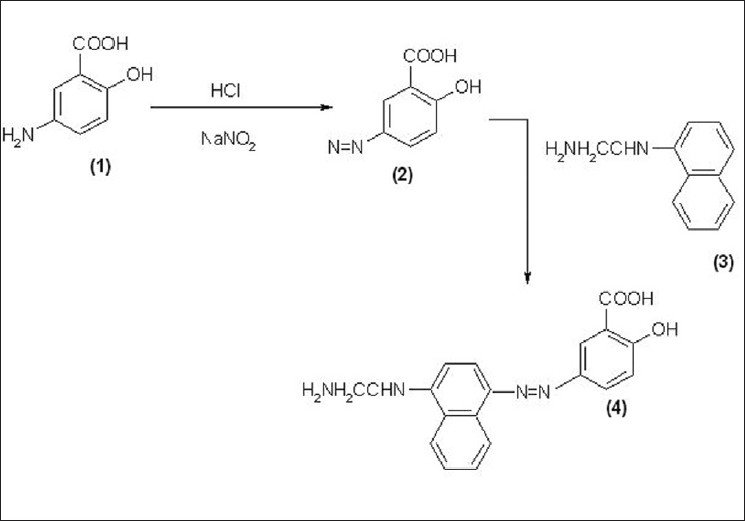
The reaction mechanism for method A

It is based on Diazotization of Mesalamine (1) with nitrous acid, to form diazotized Mesalamine (2), followed by its coupling with N-(1-naphthyl) ethylene-diamine dihydrochloride [Bratton-Marshall reagent] (3) to form a violet colored chromogen (4) with maximum absorption at 552 nm; it obeyed the Beer’s law in the concentration range of 2 – 30 μg/ml. The reaction mechanism for method A is shown in [Figure 1].

### Method B

**Figure 2:**
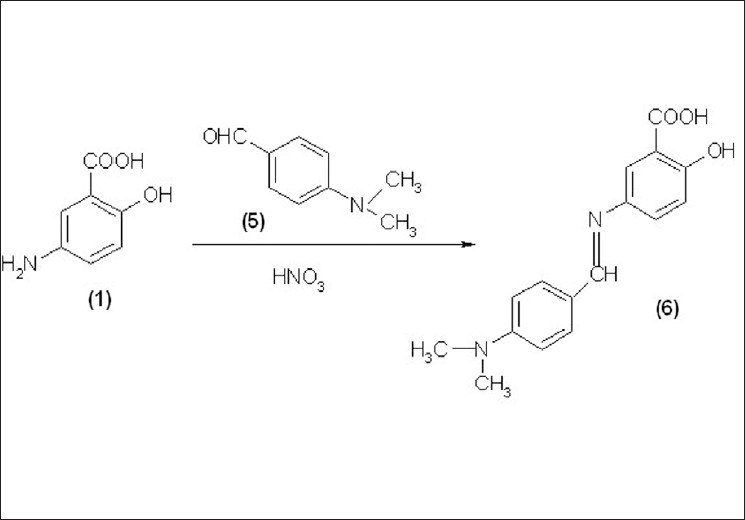
The reaction mechanism for method B

It is based on the condensation of Mesalamine (1) with p-dimethylaminobenzaldeyde (5) to form the Schiff’s base (6) that is a yellow colored chromogen and shows maximum absorbance at 440 nm; The Beer’s law is obeyed in the concentration range of 50 – 500μg/ml. The reaction mechanism for method B is shown in [Figure 2].

### Method C

**Figure 3:**
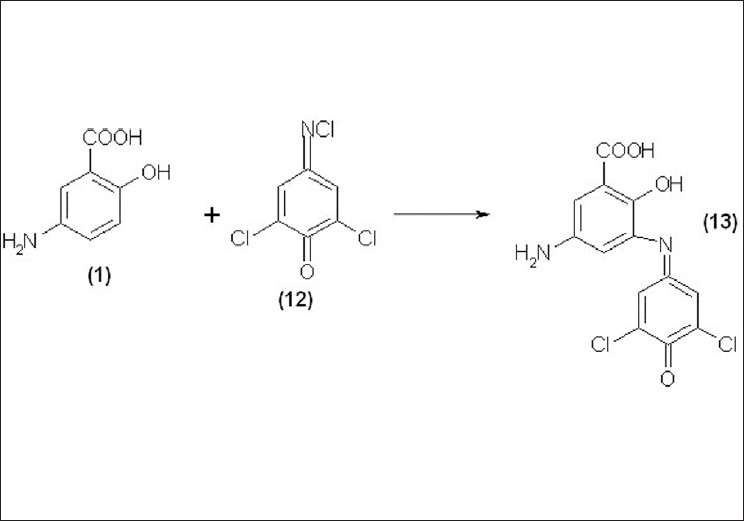
The reaction mechanism for method C

Mesalamine (1) has a phenolic group when made to react with Gibb’s reagent (12), in alkaline pH it forms a colored chromogen (13), exhibiting absorption maximum at 494 nm, and Beer’s law is obeyed in the concentration range of 5 – 60 μg/ml. The reaction mechanism for method C is shown in [Figure 3].

Spectrophotometric parameters are established for the standardization of the methods including statistical analysis of the data. These methods have been successfully extended to tablets containing mesalamine.

A Shimadzu UV / Visible double beam spectrophotometer (model 1700), with 1 cm-matched quartz cells, was used for all spectral measurements. Scanning range of 190 – 380nm for UV range and 380 – 800nm for visible range were used. All chemicals used were of A.R. grade from S.D. Fine Chemicals, Mumbai. Mesalamine was kindly gifted by Sun Pharma Ltd, Dadra.

Mesalamine equivalent to 100 mg was weighed accurately and dissolved in 2 ml of concentrated HCl in a 100 ml volumetric flask, and diluted up to the mark with water (1 mg/ml) for visible spectrophotometric determination of mesalamine, using BMR (0.2%), HCL (2N), sodium nitrite (0.3%), and ammonium sulfamate (0.1%).

Twenty tablets of mesalamine were weighed and powdered. Powder equivalent to 100 mg mesalamine was weighed and mixed with 2 ml of concentrated HCl and 75 ml of distilled water. The mixture was shaken occasionally and filtered through Whatman No.41 filter paper. The residue was washed thoroughly with water. The filtrate and the washing were combined and the final dilution was brought up to the 100 ml mark in a 100 ml volumetric flask with distilled water. In case of formulation, one brand of commercially available tablets (Mesacol) was analyzed by the proposed methods.

## PROCEDURE

### Method A

**Figure 4:**
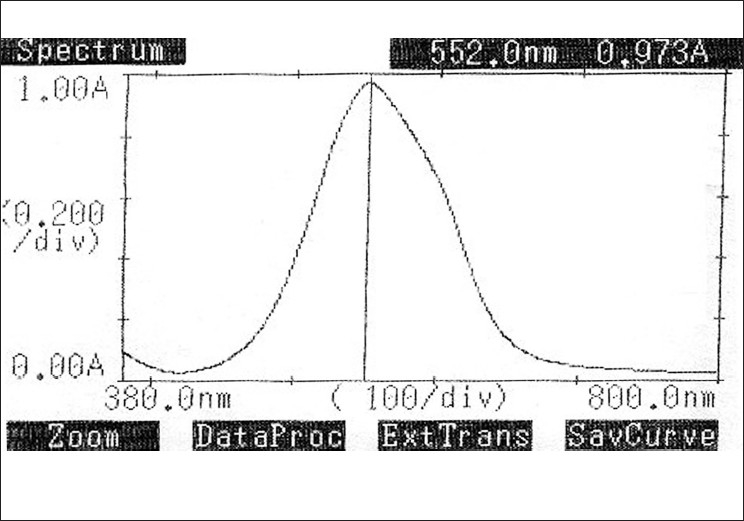
Absorption Spectrum of Mesalamine with Bratton-Marshall reagent

Aliquots of standard solution of mesalamine ranging from 0.5 – 3 ml (1 ml = 100 μg) were transferred into a series of 10 ml volumetric flasks. To each flask, 1.0 ml of hydrochloric acid (2 N) and 1.0 ml of sodium nitrite (0.3% w/v) were added and a reaction time of 10 minutes at 0 – 5°C was given for completion of the reaction. Next, 1.0 ml of ammonium sulfamate (0.1% w/v) was added to each flask with gentle shaking and after 1 minute, 1 ml of BMR reagent (0.2%w/v) was added, and kept for 20 minutes. Finally the volume in each flask was brought up to the 10 ml mark with distilled water. The absorbances of violet-colored chromogen were measured at 552 nm against the reagent blank Figure 4. The colored chromogen was stable for one hour. The amount of mesalamine present in the sample solution was computed from the respective calibration curve.

### Method B

**Figure 5:**
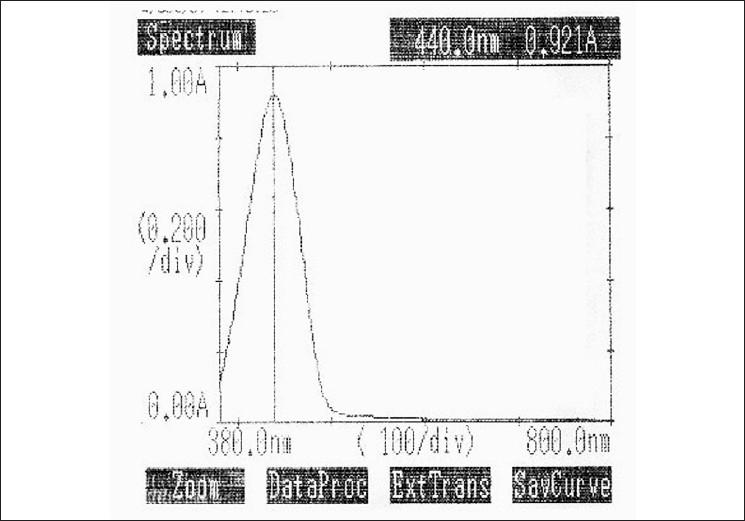
Absorption Spectrum of Mesalamine with PDAB reagent

Aliquots of standard solution of mesalamine ranging from 0.5 to 5 ml (1 ml = 1000 μg) were transferred into a series of 10 ml volumetric flasks. To each flask, 2 ml of ethanolic PDAB and 1 ml of nitric acid (4 N) were added. The solutions were warmed on a water bath for 10 minutes at 60 – 70°C. The resulting solutions were cooled to room temperature and the volume was brought up to the mark with water. The absorbances of the light yellow colored chromogen were measured at 440 nm against the reagent blank Figure 5. The colored chromogen was stable for three hours. The amount of mesalamine present in the sample solution was computed from the respective calibration curve.

### Method C

**Figure 6:**
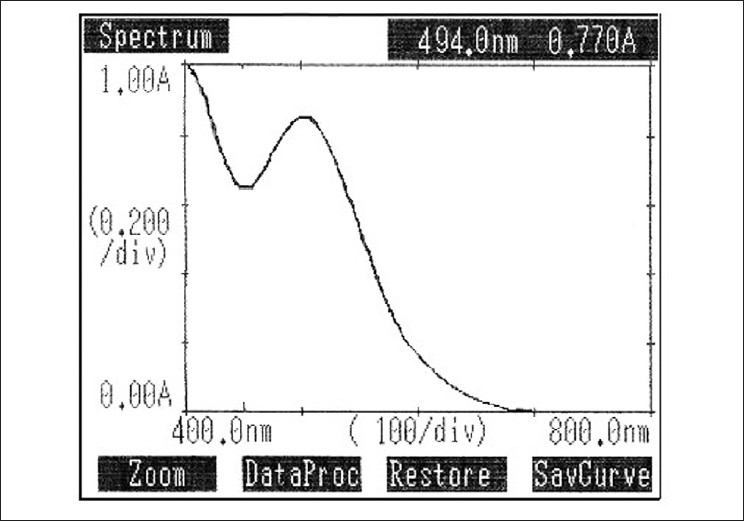
Absorption Spectrum of Mesalamine with Gibb’s reagent

Aliquots of standard solution of mesalamine ranging from 0.5 – 6 ml (1 ml = 100 μg) were transferred into a series of 10 ml volumetric flasks. To each flask, 0.5 ml of Gibb’s reagent (0.2%w/v in ethanol) and 1.0 ml of sodium nitrite (0.5% w/v) was added. The volume in each flask was brought up to 10 ml with distilled water. The absorbances of colored chromogen were measured at 494 nm against the reagent blank Figure 6. The colored chromogen was stable for eight hours. The amount of mesalamine present in the sample solution was computed from the respective calibration curve.

The optical characteristics such as absorption maxima, Beer’s law limits, molar absorptivity, and Sandell’s sensitivity are presented in [Table 1]. The regression analysis using the method of least squares was performed for the slope (b), intercept (a), and correlation (r) obtained from different concentrations and the results are summarized in [Table 1]. The percentage relative standard deviation and percentage of error (0.05 and 0.01 level of confidence limit) were calculated from the mean of eight determinations. Beer’s law limits of mesalamine are given in [Table 1]. The results showed that these methods had reasonable precision. Comparison of the results obtained with the proposed and UV methods for the dosage forms [Table 2] confirmed the suitability of the methods for pharmaceutical dosage forms when compared with the UV method. The proposed methods were reaction-specific and eliminated interference from impurities. The optimum conditions for color development for methods A, B, and C were established by varying the parameters one at a time, keeping the other parameters fixed, observing the effects of the product on the absorbance of the colored species, and incorporating them in the procedures.

**Table 1:** Optical Characteristics and Precision

Parameters	BMR	PDAB	Gibbs
Xmv (nm)	552	440	494
Beer’s law limits (μg/ml) (C)	2-30	50-500	May-60
Molar absoiptivity (L mol^−1^ cm^−1^)	6.237×102	3.744×102	1.959×103
Sandell’s sensitivity (μg/cm^2^−0.001 absorbance unit)	0.0249	0.415	0.007
Regression equation (Y*)	0.0391	0.0237	0.0168
Slope (b)			
Intercept (a)	0.0003	0.0041	0.0074
Correlation coefficient(r)	0.997	0.998	0.9988
% RSD	0.0012	0.00436	0.143
Range of errors**	0.00038	0.0017	0.0006
Confidence limits with 0.05 level			
Confidence limits with 0.01 level	0.00056	0.0026	0.0009
Limit of detection	0.04	0.699	0.1712
Limit of quantitation	0.116	1.84	0.4505

**Table 2:** Evaluation of Mesalamine in Pharmaceutical Preparations

Sample (Tablet)	Labelled amount (mg)	Amount obtained* (mg)		Percentage recovery**
		Proposed method	Reference method	
		BMR	UV	BMR
Tt	400	398.14±0.02	399.58±0.02	99.49±A.05
		PDAB	uv	PDAB
Tt	400	399.92±0.02 Gibb’s	399.58±0.02 UV	99.46±.03 Gibb’
		Gibb’s	UV	Gibb’
Tt	400	399.92±0.02	399.58±0.02	99.46±0.03

* Average of five determinations

** Mean and Standard deviation of eight determinations (100 mg of Mesalamine was added and recovered)

To evaluate the validity and reproducibility of the methods, known amounts of the pure drug were added to the previously analyzed pharmaceutical preparations and the mixtures were analyzed by the proposed methods. The percent recoveries are given in [Table 2]. Interference studies revealed that the common excipients and other additives such as lactose, starch, gelatin, talc, and magnesium trisilicate did not interfere at their regularly added levels.

## RESULT

Three new, sensitive and most economical analytical colorimetric methods were developed for the estimation of Mesalamine in bulk and pharmaceutical dosage forms. These methods are validated in terms of sensitivity, accuracy, and precision. The results were found to be accurate, and free from the interference of tablet excipients. The active pharmaceutical ingredient was extracted from its completed dosage form using hot water and HCl. The % recovery, linearity, and range, LOD and LOQ, Sandell’s sensitivity and molar absorptivity for mesalamine are summarized in [Tables 1 and 2]. The reaction mechanisms for all methods are shown in Figures 1–3.

## CONCLUSION

The UV spectroscopic methods and colorimetric methods demonstrated herein, are applicable to the estimation of Mesalamine in the pure as well as the existing dosage forms. In order to ensure that the data is generated, the above-mentioned methods would prove both accurate and precise. The experiments have been performed on calibrated equipments using suitable reference standards. To prove and document their reliability, the methods have been carried out to a possible extent. The results expressed in [Tables 1 and 2] are for Spectrophotometric methods. In addition to the positive requirements for analytical methods, the striking advantage of all the presently developed methods is that they are economical.

The proposed methods are found to be simple, sensitive, selective, accurate, precise, and economical, and can be used in the determination of mesalamine in bulk drug and its pharmaceutical dosage forms tablets in a routine manner.
